# Novel echocardiographic techniques to assess left atrial size, anatomy and function

**DOI:** 10.1186/1476-7120-10-4

**Published:** 2012-02-01

**Authors:** Matteo Cameli, Matteo Lisi, Francesca Maria Righini, Sergio Mondillo

**Affiliations:** 1 Department of Cardiovascular Diseases, University of Siena, Italy

**Keywords:** Echocardiography, Left atrium, Three-dimensional echocardiography, Speckle tracking, Strain

## Abstract

Three-dimensional echocardiography (3DE) and speckle tracking echocardiography (STE) have recently applied as imaging techniques to accurately evaluate left atrial (LA) size, anatomy and function. 3DE and off-line quantification softwares, have allowed, in comparison to magnetic resonance imaging, the most time-efficient and accurate method of LA volume quantification. STE provides a non-Doppler, angle-independent and objective quantification of LA myocardial deformation. Data regarding feasibility, accuracy and clinical applications of LA analysis by 3DE and STE are rapidly gathering. This review describes the fundamental concepts of LA 3DE and STE, illustrates how to obtain respective measurements and discuss their recognized and emerging clinical applications.

## Background

The progress of echocardiography has gone hand in hand with the growth of knowledge of the function and role of the left atrium in cardiovascular disease. Echocardiography started from information about the shape and size of the atrium: first with M-mode technique, then with two-dimensional measurement of the area and then with the assessment of atrial systolic volumes. However, even though the clinical importance of left atrial (LA) size and function has always been well known, its assessment has been neglected for some time due to the lack of tools able to assess a complete evaluation of its anatomy and performance. Recent population-based studies have demonstrated the prognostic value of LA analysis for long-term outcome. In fact, LA structural and functional remodeling has been proposed as a barometer of diastolic burden and a predictor of common cardiovascular outcomes such as atrial fibrillation, stroke, congestive heart failure, and cardiovascular death [1-3]. The structural and functional analysis of LA reflects a spectrum of pathophysiological changes that have occurred in response to specific stressors; in fact, the left atrium is exposed directly to left ventricular diastolic pressure through the open mitral valve and because of its thin wall structure it tends to reduce its elastic properties and finally to dilate with increasing pressure.

Herein, we present an overview of two novel echocardiographic techniques, three-dimensional echocardiography (3DE) and speckle tracking echocardiography (STE) [4], that have evolved recently and have conferred new insight into LA structural and functional mechanics, paying attention to describe the technological aspects and the advantages and limitations of both these techniques.

## Three-dimensional echocardiography

LA size assessment represents a significant predictor of morbidity and mortality in many cardiovascular conditions [5,6]; it has been shown recently that indexed LA volume is a more robust cardiovascular risk marker than LA area or diameter [7].

Even if 2D echocardiography remains the current standard in clinical practice for the assessment of LA size [8], the use of real-time three-dimensional echocardiography (RT3DE) has been recently introduced as a new technique for the assessment of LA volume and LA ejection fraction (LAEF).

### Methods

LA volumes by RT3DE is collected in four-cycles full-volume made during a breath hold, using a 3D matrix-array transducer. Acquisition is triggered to the electrocardiographic R wave. Particular care is taken to ensure that the entire LA is included within a pyramidal 3D data set. The pyramidal volume data is displayed in three different cross-sections that may be modified interactively by manual shifting of vertical and horizontal lines in the two orthogonal apical and the short-axis views. LA volume by 3D echocardiography is derived from semi-automated tracing of the LA endocardium, starting the measurements in the frame with the largest atrial dimension, corresponding to ventricular end-systole, just before opening of the atrio-ventricular valves, in perpendicular apical long-axis planes.

This is performed by making 5 points in the atrial surfaces of the mitral annulus: at the anterior, inferior, lateral, and septal annulus, and the fifth point at the apex of the left atrium. Following this manual identification, the program automatically identifies the endocardial surface using a deformable shell model [9,10]. Then manual adjustment of the endocardial surface is performed in all examinations presented, in order to include trabeculae and to exclude atrial appendages and large veins from the cavity volumes. Then the frame with the smallest atrial dimension (at ventricular end-diastole) is selected with similar surface detection and manual editing.

**Figure 1:**
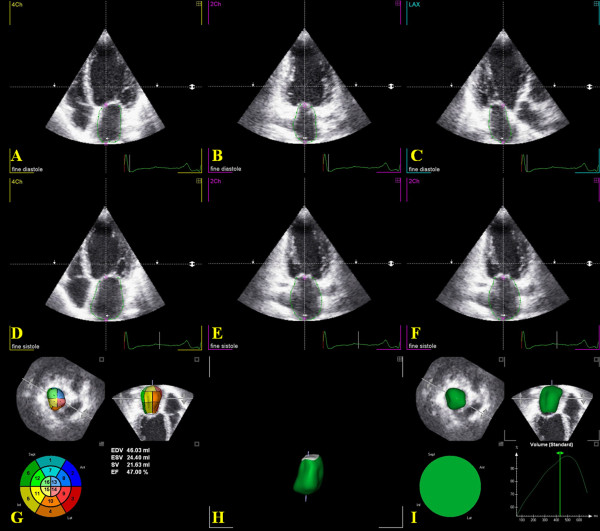
**An example of three-dimensional echocardiographic reconstruction of LA volume (ml) and LA ejection fraction (LAEF%) measurements by TomTec software, after the acquisition of a full-volume dataset**. Delineation of the endocardial border at end-diastole (A-C) and end-systole (D-F), estimation of LAEF (G), LA volume reconstruction (H) and LA time-volume curves (I).

Atrial maximum (max) and minimum (min) volumes are obtained, and atrial EF is derived from the two volumes (Figure 1).

Recently Aune et al. [11] have reported the normal reference values of LA, right atrial (RA) volumes, and LAEF obtained by 3DE in one hundred and sixty-six healthy subjects, aged 29-79 years. In this study upper normal values for LA volume and LAEF were similar for both genders; RA volumes was 15% higher than LA ones (Table 1).

**Table 1:** Reference ranges of left and right atrial indexed volumes (LAVI and RAVI) and of ejection fractions of both atria (LAEF and RAEF) obtained with real-time three-dimensional echocardiography (RT3DE).

RT3DE	Males (n = 75)	Females (n = 84)	Total Study Population (n = 159)
**LAVI max **(ml/m^2^)	15-42	15-39	15-41
**LAVI min **(ml/m^2^)	6-20	5-18	5-19
**LAEF **(%)	46-77	44-80	45-79
**RAVI max **(ml/m^2^)	18-50	17-41	18-47
**RAVI min **(ml/m^2^)	7-22	5-18	5-20
**RAEF **(%)	46-74	48-83	46-80

[Modified from Aune et al. Eur J Echocardiogr 2009;10:738-44]

### Advantages and limitations

LA volume measurements by RT3DE correlate closely with those obtained on multidetector computed tomography [10] and on magnetic resonance imaging [12,13], showing a better diagnostic accuracy respect to 2D methods [14].

In particular, 3DE reconstruction overcomes 2DE limitations, avoiding geometric assumptions for LA volume evaluation [15] (Figure 1). The latest generation of 3D matrix-array transducers and off-line quantification softwares, has allowed, in comparison to MRI, the most time-efficient method of LA volume quantification, with the exception of poor image quality or cardiac arrhythmias [13].

**Figure 2:**
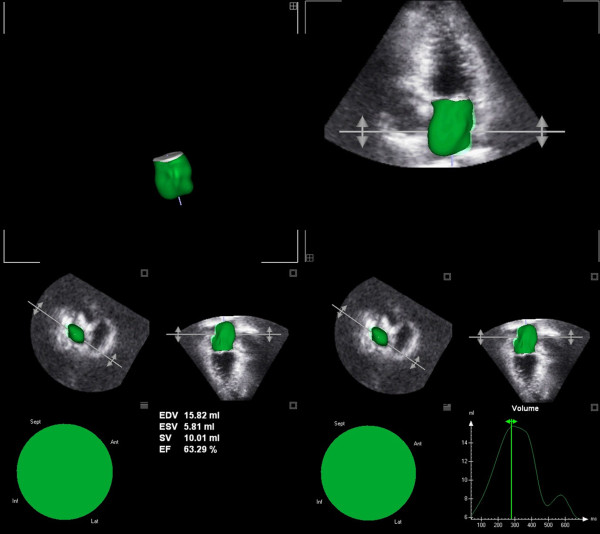
**Three-dimensional echocardiographic LA reconstruction in a healthy subject: Normal LA volume and normal LAEF**.

**Figure 3:**
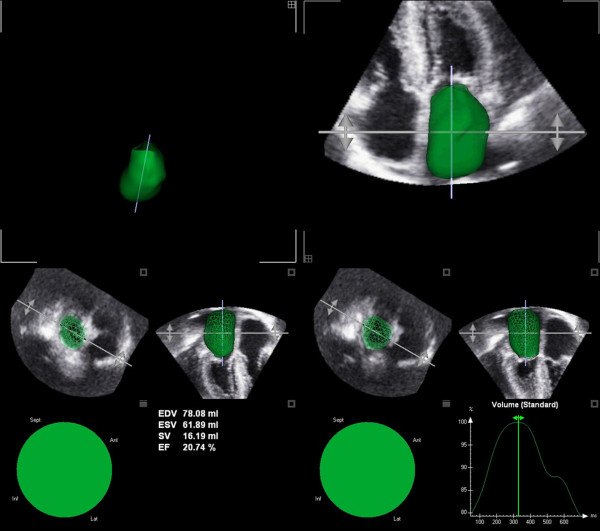
**Three-dimensional echocardiographic LA reconstruction in a patient affected by cardiac amylodosis: LA enlargement and reduced LAEF**.

Moreover, through 3DE it is possible to estimate LA ejection fraction (LAEF) (Figure 2); despite recent guidelines did not mention any possible application, several clinical conditions may require three-dimensional LA function analysis (Figure 3). Recent reports have shown potential applications of LAEF in different patterns of diastolic dysfunction, demonstrating a good correlation with the E/E' ratio [16].

Despite the good correlation between 3DE and MRI in estimating LA volume, several studies show that echocardiography systematically tends to underestimate LA volumes when compared to MRI [17,18]. A probable cause of this disparity is the difference in spatial image resolution between both 2D and 3DE compared to MRI; in fact echocardiography presents low lateral image resolution because apical window places the left atrium at the far field of the ultrasound beam. In addition 2D and 3D ultrasound images could not be able to distinguish the volumes within the intratrabecular atrial areas [13].

Moreover, as with any new imaging technique, a learning curve exists, and recognizing and avoiding potential artifacts are critical and most of all related to respiratory or ECG gating and/or incorrect gain settings which are particular challenging in patients with arrhythmias or respiratory difficulties [19].

### Clinical applications

3D echocardiography is a safe, non-invasive imaging modality that may be a very promising tool to assess atrial size, providing important insights into LA morphologic and functional evaluations.

Recently, it has been shown that LA volume provides a more accurate measure of LA size than conventional M-mode LA dimension [20]. In fact LA enlargement occurs in all 3D directions but not in a uniform fashion and medial-lateral expansion is less prominent than longitudinal and antero-posterior expansions, so one-dimensional assessment is likely to be an insensitive assessment of any change in LA size [21]. For these reasons, the ASE has recommended quantification of LA size by biplane 2D echocardiography.

However, cyclic changes in LA volume may not be observed directly by two-dimensional echocardiography because the shape of the left atrium changes during the heart cycle and generally only maximum LA volume is routinely measured in clinical practice.

The physiologic volume changes that occur in the different phases of cardiac cycle may be accurately evaluate by RT3DE; real-time acquisition of 3-dimensional views during a routine echocardiographic examination allows us to analyze the dynamic changes in LA volume and to quantify the contribution of LA to LV filling, calculating also LA ejection fraction by the 3D semi-automated software [16].

The relative contribution of LA phasic function to LV filling is dependent upon the LV diastolic properties; with increased stiffness or noncompliance of the LV, LA pressure rises to maintain adequate LV filling, and the increased atrial wall tension leads to chamber dilatation and stretch of the atrial myocardium [20]. Thus, LA volume increases with severity of diastolic dysfunction. The structural changes of the LA may express the chronicity of exposure to abnormal filling pressures and provide predictive information beyond that of diastolic function grade. In this way, LA volume reflects an average effect of LV filling pressures over time, rather than an instantaneous measurement at the time of study. Thus, Doppler and tissue Doppler assessment of instantaneous filling pressure is better suited for monitoring hemodynamic status in the short term, whereas LA volume is useful for monitoring long-term hemodynamic control [20].

## Speckle tracking echocardiography

The atrial longitudinal strain, deriving from application of the analysis of myocardial deformation using STE at atrial chambers, results the first parameter useful for functional analysis of the LA and, as shown by recent studies, it presents considerable feasibility and reproducibility [22-25].

### Methods

Two-dimensional strain imaging is an echocardiographic technique that uses standard B-mode images for speckle tracking analysis. The speckle pattern (acoustic backscatter generated by the reflected ultrasound beam) is followed frame-by-frame, using a statistical approach based on the detection of the best matching area. The displacement of this speckled pattern is considered to follow myocardial movement and a change between speckles is assumed to represent myocardial deformation [4].

Although this new technique was introduced for the exclusive analysis of left ventricular (LV) function, several studied have recently extended its applicability to other cardiac chamber, such as the left atrium [22,23,26-29].

For speckle tracking analysis of left atrial chamber, apical four- and two-chamber views images are obtained using conventional two-dimensional gray scale echocardiography, during breath hold with a stable ECG recording. Particular attention is given to obtain an adequate gray scale image, allowing reliable delineation of myocardial tissue and extracardiac structures. Three consecutive heart cycles are recorded and averaged. The frame rate is set between 60 and 80 frames per second; these settings are recommended to combine temporal resolution with adequate spatial definition, and to enhance the feasibility of the frame-to-frame tracking technique [22].

Recordings are processed using an acoustic-tracking software (Echo Pac, GE, USA), allowing off-line semi-automated analysis of speckle-based strain.

In particular, LA endocardial surface is manually traced in both four- and two-chamber views by a point-and-click approach. An epicardial surface tracing is then automatically generated by the system, thus creating a region of interest (ROI). To trace the ROI in the discontinuity of LA wall corresponding to pulmonary veins and LA appendage, the direction of LA endocardial and epicardial surfaces at the junction with these structures is extrapolated. After manual adjustment of ROI width and shape, the software divides the ROI into 6 segments, and the resulting tracking quality for each segment is automatically scored as either acceptable or non-acceptable, with the possibility of further manual correction. Segments in which no adequate image quality can be obtained are rejected by the software and excluded from the analysis. In subjects with adequate image quality, a total of 12 segments are then analyzed [22].

**Figure 4:**
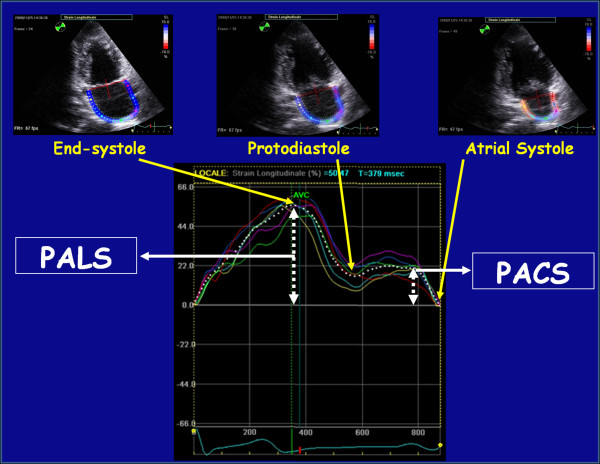
**Peak atrial longitudinal strain (PALS) and peak atrial contraction strain (PACS) in a representative subject**.

Lastly the software generates the longitudinal strain curves for each segment and a mean curve of all segments that reflect the pathophysiology of atrial function (Figure 4). During the reservoir phase, the LA fills up, stretches itself, and for this reason, the atrial strain increases, reaching a positive peak at the end of atrial filling, before the opening of the mitral valve; after the opening of the mitral valve, LA empties quickly, shortens, the strain decreases, up to a plateau corresponding to the phase of diastasis, followed by a second positive peak, but less than the first, which corresponds to the period preceding the atrial contraction, and finally a negative peak after the atrial contraction (Figure 4) [22]. This second positive deflection of the atrial strain curve, corresponding to atrial systole, is present only if the subject analyzed presents sinus rhythm. Thus, as shown in Figure 4, peak atrial longitudinal strain (PALS), measured at the end of the reservoir phase, and peak atrial contraction strain (PACS), measured just before the start of the active atrial contractile phase, are calculated by averaging values observed in all LA segments (global PALS and PACS), and by separately averaging values observed in four- and two-chamber views (four- and two-chamber average PALS and PACS, respectively). LA contraction strain index (CSI), representing, in percentual values, the contribution of the LA active contraction to the LV filling phase, is calculated as (global PACS/global PALS) × 100. The time to peak longitudinal strain (TPLS) is also measured as the average of all 12 segments (global TPLS) and by separately averaging values observed in the two apical views (four- and two-chamber average TPLS) [30].

Numerous methodological studies have shown the reference range values and good feasibility and reproducibility of the application of speckle tracking technique to measure LA myocardial longitudinal deformation (Table 2) [22-25]. Regarding the measurement of peak atrial strain, as stated in the current ASE/EAE Consensus [31], two techniques have been proposed to quantify atrial deformation by STE, which differ only by the choice of frame from which start processing software.

**Table 2:** Reference values of global, 4-chamber, and 2-chamber peak atrial longitudinal strain (PALS) and time to peak strain (TPLS).

	Mean ± DS	5°-95° Percentile
**PALS **(%)		
Global	42.2 ± 6.1	32.2 - 53.2
4-chamber	40.1 ± 7.9	29.0 - 53.6
2-chamber	44.3 ± 6.0	35.2 - 52.7
**TPLS **(ms)		
Global	368.0 ± 29.9	322.9 - 430.4
4-chamber	364.2 ± 42.6	300.8 - 436.9
2-chamber	367.4 ± 34.1	326.4 - 435.2

The first takes as reference point the QRS onset and measures the positive peak atrial longitudinal strain, corresponding to atrial reservoir, the second uses the P wave as the reference point, enabling the measurement of a first negative peak atrial longitudinal strain, corresponding to atrial systole, a second positive peak atrial strain, corresponding to LA conduit function, and their sum.

### Advantages and Limitations

STE analysis allows an excellent assessment of atrial deformation profile during an entire cardiac cycle, closely following LA physiology [22]. Considering the limitations of classical indices of LA function, assessment of LA strain by speckle tracking may represent a relatively rapid and easy-to-perform technique to explore LA function, due to its semi-automated nature and to its off-line processing. In fact, in contrast to Doppler-derived parameters, speckle tracking has the advantage of being angle-independent, and to be less affected by reverberations, side lobes and drop out artefacts [22].

Nonetheless, intrinsic limitations of speckle tracking include strict frame rate dependency, potential errors in epicardial/endocardial border tracing in subjects with suboptimal image quality, and need for an appropriate learning curve to achieve adequate experience in using analysis software. In fact, the potential difficulty of accurately obtaining a region of interest close enough to the effective shape of the left atrium, and the risk of contamination by signal components arising from structures surrounding the left atrium should be considered [22].

Lastly, because a dedicated software for LA strain analysis has not yet been released, the analysis is performed using a software created for the left ventricle; for this reason, it is mandatory to be careful in the endocardial border delineation, excluding from the analysis the auricola and the outlet of the pulmonary veins, in order to minimize the risk of artefacts caused by signals from these structures [4].

### Clinical applications

The LA mechanical function studied by STE can be described by three phases, in which the atrial strain curve closely follow LV dynamics during the cardiac cycle [22]. In fact during the period of LA reservoir, corresponding to the phases of LV isovolumic contraction, ejection, and isovolumic relaxation, LA strain increases, achieving a peak at the end of LA filling, due to the downward movement of the mitral annulus towards the apex, as a result of LV contraction. In the same way, during the conduit and LA contraction phases, the LA strain curve reflects inversely the pattern of LV myocardial deformation. Therefore LA function seems to be influenced not only by LA stiffness but also by LV compliance during ventricular filling and by contraction through the descent of the base during LV systole [32].

It has been recently demonstrated that this new approach for the study of LA function is of potential clinical importance in a number of pathophysiologic conditions typically associated to abnormal LA function, e.g. mitral valve diseases, supraventricular arrhythmias, systemic hypertension, ischemic heart disease, heart failure, atrial stunning, and cardiomyopathies [22].

In this overview, we have decided to focus on two of these pathologic conditions in which atrial strain imaging may be helpful in clinical management of patients.

#### Diastolic Function

Patients with diastolic heart failure (DHF) suffer from increased morbidity and mortality. Although advanced age, hypertension, diabetes, coronary heart disease and female gender identify patients at high risk of DHF, the underlying pathophysiological mechanisms for the transition from an asymptomatic state to a state of symptomatic heart failure are not well defined [33,34].

**Figure 5:**
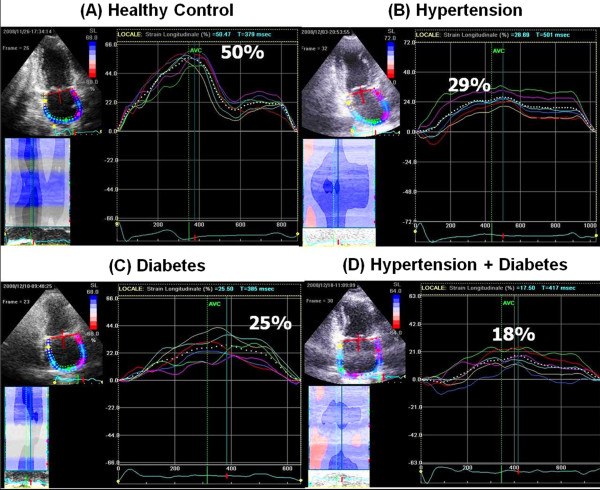
**Peak atrial longitudinal strain measurements in a healthy subject (A) and in a hypertensive (B), diabetic (C) and hypertensive-diabetic (D) patients**. Atrial strain appeared reduced in hypertensive and in diabetics. In the case of association of the two diseases the reduction is even more evident.

A study performed by measuring the Doppler strain rate atrial performance revealed that a reduction of atrial strain rate is able, in contrast to the atrial ventricular mass and volume, to differentiate patients with DHF than patients with simple diastolic dysfunction [35]. Using STE, it has been demonstrated in hypertensive patients, diabetic and hypertensive-diabetic patients with ejection fraction and LA volumes preserved, that the atrial strain occurs progressively reduced, demonstrating the ability of this new method in identifying the premature atrial dysfunction before the appearance of LA structural changes, identified also by standard methods (atrial dilatation) (Figure 5) [36]. In addition, a study in hypertensive patients at an early stage, with no evidence of increased ventricular mass, showed early alterations of atrial strain even before the development of diastolic dysfunction [37]. Even in athletes it has been recently shown a particular LA strain pattern [38].

The analysis of atrial strain has resulted very useful also to other clinical settings, as in end-stage heart failure. In these patients it is essential the management of the precarious hemodynamic balance and the accurate estimation of left ventricular filling pressure is very useful to assure proper management. The ratio E/E', the main parameter for echocardiographic estimation of ventricular filling pressures, was recently proved inadequate for this purpose [39]. Atrial longitudinal strain has instead demonstrated a good correlation and diagnostic performance in an accurate estimation of high pulmonary capillary wedge pressure (> 18 mmHg) [40,41]; greater left ventricular filling pressure is associated to a reduced left atrial deformation.

#### Atrial Fibrillation

**Figure 6:**
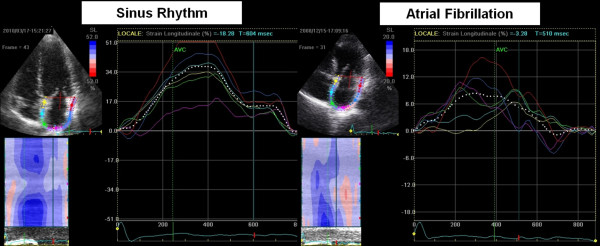
**Comparison of peak atrial longitudinal strain in a healthy subject (*left*) and one patient with atrial fibrillation (*right*)**. Note in the patient with atrial fibrillation the disappearance of the second deflection of the curve, relative to the atrial contraction phase.

**Figure 7:**
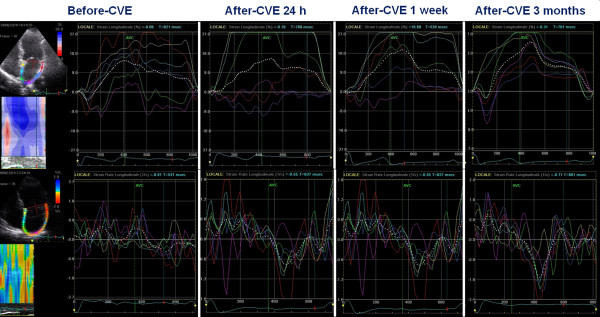
**Evaluation of left atrial strain and strain rate before and after electrical cardioversion**. Note the progressive improvement of the peak strain and the reappearance of the second deflection of the atrial strain curve, relative to the atrial contraction phase.

**Figure 8:**
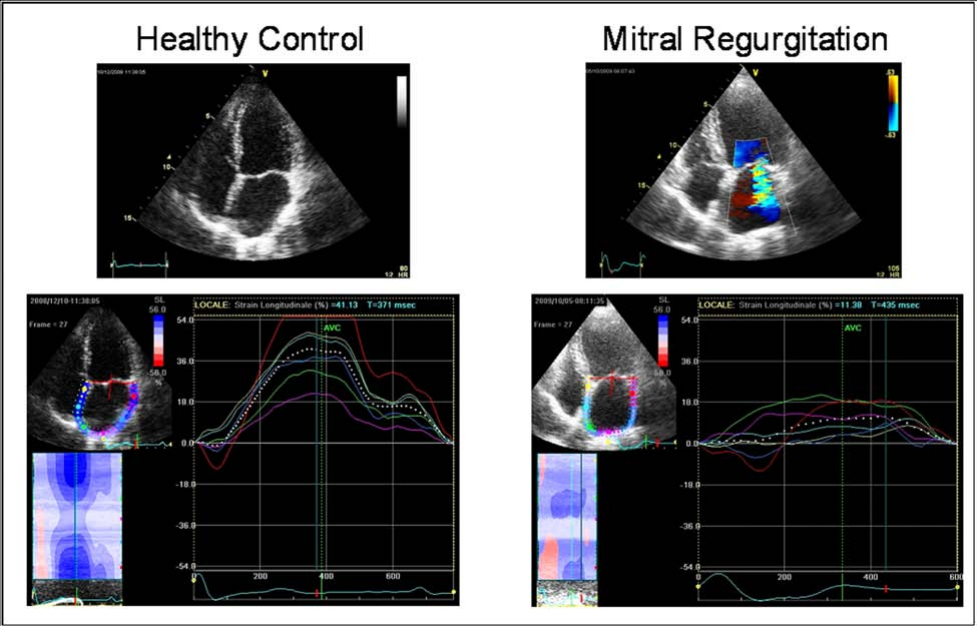
**Peak Atrial Longitudinal Strain (PALS): two representative cases of an healthy control (left) and of a patient with severe mitral regurgitation (right)**.

**Figure 9:**
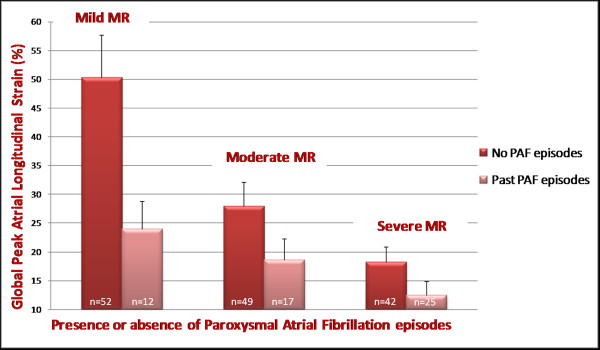
**Relation of global peak atrial longitudinal strain (PALS) to the presence or absence of paroxysmal atrial fibrillation (PAF) episodes, cross-tabulated by category of each underlying mitral regurgitation severity**.

From the pathophysiological point of view this arrhythmia is related to the remodelling and atrial dilatation, as determined by the proliferation and differentiation of fibroblasts into myofibroblasts and by the increase of connective tissue resulting in the appearance of fibrosis. This structural remodelling determines dissociation between muscle bundles and electrical alterations in the conduction of electrical stimulation, which would facilitate the initiation and maintenance of this arrhythmia. For this reason it is very useful to analyze the alterations in the function of the left atrium that can potentially help us in the study of this rhythm disorder. In patients with chronic AF often dysfunction and dilatation of the left atrium are present. Through the analysis of strain of the LA, it appears that the peak strain of atrial contraction is absent (Figure 6); recent evidences have demonstrated that lower PALS correlated with cerebral stroke events [42,43]; PALS is reduced after electrical cardioversion or ablation of the sinus node, while it increases gradually in subsequent months (Figure 7). Several studies have shown that the strain rate curve of the left atrium is impaired in patients with AF. The peak systolic strain rate is reduced in patients with paroxysms of AF and chronic AF in particular, goes gradually to normalization after cardioversion [44], and is inversely related to age, LA volume [45,46], and the presence of LA wall fibrosis [47]. Even the peak of atrial contraction is reduced [48], and this value is inversely correlated with pulmonary pressures. In patients with various degrees of mitral regurgitation it has been demonstrated, in addition to a progressively reduced atrial strain (Figure 8) [30], that subjects with the same severity of valvular disease, atrial strain was significantly lower in patients who reported a history of episodes of paroxysmal atrial fibrillation (Figure 9) [49].

## Discussion

LA size and function data may fulfil the gaps left by the other conventional two-dimensional echocardiographic parameters and may allow a more complete diagnosis characterization. In fact, the evaluation of the left atrium has been until recently performed by echocardiographic purely with morphometric and static parameters, such as the anterior-posterior diameter, area and 2D volume. However, the property role of the left atrium is very important in maintaining cardiac performance [50].

Actually, real-time 3-dimensional echocardiography permits to assess the physiologic volume changes of the left atrium during cardiac cycle and to quantify the contribution of LA contraction to LV filling, through the measurement of LAEF [16].

While LA size provides information of long-term hemodynamic control, the study of atrial myocardial deformation by STE is able to improve our understanding on LA function. In fact STE allows noninvasive assessment of global LA function and regional deformation of LA walls; two-dimensional strain imaging also successfully provides LA volume curves during one cardiac cycle, from which various LA mechanical indices can be obtained and allows a direct assessment of LA contractility and passive deformation.

Several studies have shown that strain imaging can detect LA dysfunction before the manifestation of LA structural changes. The decrease of LA reservoir and the increase of LA pump functions are the first manifestations of the burden of diastolic dysfunction, appearing before the LA structural changes [51].

Considering the limitations of classical indices of LA, 3D volume and speckle strain assessment of LA may represent a relatively rapid and easy-to-perform technique to explore LA size and function.

## Conclusion

LA size and function carry important clinical and prognosis implications.

The measurement of LA volume by 3D echocardiography is superior to the other conventional 2D parameters as a measure of LA size and should be incorporate into routine clinical evaluation. Regional assessment of LA function by STE also provides more detailed information about LA mechanics and may prove to have a very important clinical impact.

## Competing interests

The authors declare that they have no competing interests.

## Authors' contributions

MC conceived the review and drafted the manuscript. ML revised critically the manuscript and added figures which resulted in a more readable manuscript and finally helped in the collection of the bibliography suggesting some important papers reported in the review and improved the chapter concerning three-dimensional echocardiography. FMR suggested the scheme of the review and revised critically the paper, contributed and improved the chapters concerning the role of the new technology of speckle tracking echocardiography. SM participated in the design of the review and gave the final approval. All authors read and approved the final manuscript.
